# Feline Infectious Peritonitis Virus Nsp5 Inhibits Type I Interferon Production by Cleaving NEMO at Multiple Sites

**DOI:** 10.3390/v12010043

**Published:** 2019-12-30

**Authors:** Si Chen, Jin Tian, Zhijie Li, Hongtao Kang, Jikai Zhang, Jiapei Huang, Hang Yin, Xiaoliang Hu, Liandong Qu

**Affiliations:** State Key Laboratory of Veterinary Biotechnology, Harbin Veterinary Research Institute, Chinese Academy of Agricultural Sciences, Harbin 150001, China; phdchensi@163.com (S.C.); tj6049345@126.com (J.T.); lizhijie@caas.cn (Z.L.); kanghongtao@caas.cn (H.K.); 15776628009@163.com (J.Z.); sicaujiapeihuang@163.com (J.H.); yinhvri@163.com (H.Y.)

**Keywords:** DF2, type I IFN, nsp5, NEMO, cleavage

## Abstract

Feline infectious peritonitis (FIP), caused by virulent feline coronavirus, is the leading infectious cause of death in cats. The type I interferon (type I IFN)-mediated immune responses provide host protection from infectious diseases. Several coronaviruses have been reported to evolve diverse strategies to evade host IFN response. However, whether feline infectious peritonitis virus (FIPV) antagonizes the type I IFN signaling remains unclear. In this study, we demonstrated that FIPV strain DF2 infection not only failed to induce interferon-β (IFN-β) and interferon-stimulated gene (ISG) production, but also inhibited Sendai virus (SEV) or polyinosinic-polycytidylic acid (poly(I:C))-induced IFN-β production. Subsequently, we found that one of the non-structural proteins encoded by the FIPV genome, nsp5, interrupted type I IFN signaling in a protease-dependent manner by cleaving the nuclear factor κB (NF-κB) essential modulator (NEMO) at three sites—glutamine132 (Q132), Q205, and Q231. Further investigation revealed that the cleavage products of NEMO lost the ability to activate the IFN-β promoter. Mechanistically, the nsp5-mediated NEMO cleavage disrupted the recruitment of the TRAF family member-associated NF-κB activator (TANK) to NEMO, which reduced the phosphorylation of interferon regulatory factor 3 (IRF3), leading to the inhibition of type I IFN production. Our research provides new insights into the mechanism for FIPV to counteract host innate immune response.

## 1. Introduction

Feline coronaviruses (FCoVs) are important pathogens of domestic cats and wild felids, including African and mountain lions [1,2]. FCoVs are classified into two pathotypes, the avirulent feline enteric coronavirus (FECV), and the virulent feline infectious peritonitis virus (FIPV). Both FECV and FIPV have two serotypes, type I and type II FCoVs, based on differences in the amino acid sequence of the S protein [3,4]. Infection with FECV is widespread among cats, especially in high-density environments, causing inapparent or mild enteritis with little clinical consequence. However, a small portion of cats develop FIP during the course of FECV infection and succumb to the disease [5]. Spontaneous mutations of the S gene [6], ORF3abc [7,8], and ORF7ab [9,10] in the FCoV genome allow virions to efficiently replicate in macrophages and monocytes, ultimately resulting in FIPV [11]. Detection of FCoV S gene mutations is considered as a tool to diagnose FIPV [12,13]. FIP is typically characterized by a fibrinous and granulomatous serositis, protein-rich serous effusions, and pyogranulomatous lesions in several organs [14,15]. Also, published reports have shown that lymphopenia in combination with massive apoptosis of uninfected T-cells which precedes the onset of clinical signs is a notable feature of both experimental and natural FIP [11,16]. Once cats develop classic clinical signs, fatality to FIP is virtually 100% [15] and the survival time from diagnosis to death is about 8–9 days [17,18]. FIPV is the leading cause of death in young cats [19], and significant research efforts still have not resulted in a fully elucidated pathogenesis.

FCoV is an enveloped, single-stranded positive-sense RNA virus, classified as an alphacoronavirus. Like all coronaviruses, FCoV replication and transcription occur in the cytoplasm, resulting not only in new genome molecules and the typical nested set of subgenomic mRNAs but also in the dsRNA intermediates [20,21,22]. The FCoV genome contains seven open reading frames (ORFs). ORF1a and ORF1b produce the coronaviral polyproteins pp1a and pp1ab. The polyproteins are then processed by virus-encoded proteases, the papain-like protease (PLpro) and the 3C-like protease (3CLpro). Since 3CLpro is structurally and functionally conservation among coronaviruses and essential for viral replication, 3CLpro is considered a potential target for anti-coronaviral drug development [23,24,25].

Interferons (IFNs) are crucial antiviral cytokines in innate immune responses. Viral infection triggers host innate immune responses through activation of the transcription factors nuclear factor κB (NF-κB) and interferon regulatory factor 3 (IRF3), which coordinately regulate the expression of type I interferons such as interferon-β (IFN-β) [26,27]. The type I interferons activate the JAK–STAT pathway to stimulate the expression of interferon-stimulated genes (ISGs), which collectively inhibit viral replication and assembly [28]. The NF-κB essential modulator (NEMO), as a regulatory protein, is an essential component of the IκB kinase complex (IKK) [29]. NEMO contains many distinct domains, including a TRAF family member-associated NF-κB activator (TANK)-binding domain [30], two coil–coiled domains (CC1 and CC2), a leucine zipper region, and a zinc finger domain [31]. Previous studies demonstrated that NEMO but not NF-κB activity is essential for virus-induced activation of IRF3 and interferon regulatory factor 7 (IRF7). NEMO interacts with TANK to recruit TANK-binding kinase 1 (TBK1) and inhibitor-κb kinase ε (IKKε) to the retinoic acid-inducible gene I (RIG-I)–mitochondrial antiviral signaling (MAVS) complex in the process of IRF3 and IRF7 activation [32]. During coevolution with their hosts, many viruses have evolved redundant mechanisms to counteract the host immunity for optimal viral adaption. Accumulating evidence shows that coronaviruses have abilities to evade host IFN response. Previous studies have shown that PEDV nsp1 is the most potent IFN antagonist. PEDV nsp1 interrupted the enhanceosome assembly of IRF3 and CREB-binding protein (CBP) by degrading CBP in a protease-dependent way [33]. SARS-CoV PLpro efficiently inhibits activation of the IRF3 pathway by disrupting the interaction between the components in stimulator of interferon genes (STING)–tumor necrosis factor receptor-associated factor 3 (TRAF3)–TBK1 [34]. SARS-CoV nsp14 is an exoribonuclease that is capable of preventing IFN responses by a specific digestion of dsRNA and subsequent removal of RNA-PAMPs [35]. MERS nsp16 is essential for interferon resistance and viral pathogenesis [36]. However, not much is known about how FIPV acquires mechanisms to evade host immune responses, except for the identification of accessory protein 7a as a counteragent against IFN-α-induced antiviral response [37]. Here, we demonstrated that FIPV strain DF2 antagonizes type I IFN signaling. We found that FIPV nsp5 as a negative regular inhibited type I IFN production by cleaving multiple sites of NEMO. Mechanistically, the FIPV nsp5-mediated NEMO cleavage disrupted the interaction between TANK and NEMO, which resulted in inhibition of IRF3 phosphorylation and suppression of type I IFN production. Our findings reveal a novel mechanism of FIPV to evade host innate immune response.

## 2. Materials and Methods

### 2.1. Cells, Virus, Antibodies

CRFK cells and HEK293T cells (American Type Culture Collection, ATCC) were grown in Dulbecco’s modified Eagle medium (DMEM; Gibco, Thermo Fisher Scientific, Waltham, MA, USA) containing 8% fetal bovine serum (FBS). The cells were incubated at 37 °C in a 5% CO2 humidified cabinet.

Antibodies, namely, mouse anti-Myc (catalogue no. Ab56), rabbit anti-Myc (catalogue no. ab9106), rabbit anti-NEMO (catalogue no. ab188569), rabbit anti-IRF3 (catalogue no. ab68481), and rabbit anti-IRF3 (phospho-S386) (catalogue no. ab76493) were purchased from Abcam. Antibodies, namely, mouse anti-HA and mouse anti-Flag (catalogue no. 62216), were purchased from Sigma. The mouse anti-*N* polyclonal antibodies were prepared by our laboratory. Briefly, the complete N gene was amplified using a forward primer (5′ TTT GGA TCC ATG GCC AAC CAG GGA CAA CGC 3′) and a reverse primer (5′ TTT GCG GCC GCTTA GTT CGT TAC CTC ATC AAT 3′). Then, the products were cloned into the vector pGEX6p-1. Purified GST-N recombinant protein was used as an antigen to inject female BALB/c mice. After three immunizations, serum was collected and stored at –80 °C. The caspase inhibitor Z-VAD-FMK, the proteasome inhibitor MG132, and the lysosome inhibitor NH4Cl were purchased from MCE.

The FIPV strain DF2 and Sendai virus (SEV) were obtained from ATCC.

### 2.2. Plasmid Construction

The feline IFN-β promoter luciferase reporter plasmid (pIFN-Luc) was described previously [38]. A pRL-TK plasmid (Promega, Madison, WI, USA) expressing the Renilla luciferase protein was used as a control. Flag-nsp5, Flag-nsp5 mutants, and HA-nsp5 were generated by cloning the ORF of nsp5 or nsp5 mutant into the p3×flag-cmv-10, pCAGGS-HA vectors, respectively. Feline NEMO constructs with an N-terminal HA tag were generated by amplification of feline NEMO cDNA and cloned into the vector pCAGGS-HA. A series of pHA-tagged NEMO mutants (NEMO-K277A, NEMOQ123A, NEMOQ132A, NEMOQ134A, NEMOQ168A, NEMOQ205A, NEMOQ207A, NEMOQ229R, NEMOQ236-239A) were cloned by overlap extension PCR using NEMO-WT as the template and constructed into pCAGGS-HA vectors. The cDNAs encoding truncated forms of NEMO, including 132N (1–132 amino acids), 132C (132–419 amino acids), 205N (1–205 amino acids), 205C (205–419 amino acids), 231N (1–231 amino acids), and 231C (231–419 amino acids), were cloned into the pCAGGS-HA vectors. The plasmids expressing feline Flag-STING, Flag-IRF3, and Flag-IRF3/5D, which were constitutively active, have been described previously [39]. The pHA-tagged feline RIG-I, MAVS, TANK, and TBK1 were constructed by using standard molecular biology techniques.

### 2.3. Dual-Luciferase Reporter Assay

CRFK cells were co-transfected with a firefly luciferase reporter plasmid IFN-β-luc at 0.2 μg/well and the Renilla luciferase reporter plasmid pRL-TK at 0.02 μg/well, in the presence or absence of expression plasmids as indicated, using Lipofectamine 2000 regent (Invitogen) according to the manufacturer’s instructions. At 24 h post-transfection, luciferase assays were conducted. The Promega luciferase assay system was used according to the manufacturer’s instructions. The data are presented as relative firefly luciferase activities normalized to Renilla luciferase activities (means ± SD) and are representative of three independent experiments.

### 2.4. Quantitative Reverse Transcription-PCR (qRT-PCR)

Total RNA was extracted using an Axygen multisource total RNA miniprep kit according to the manufacturer’s instructions. cDNA was obtained using FastKing-RT superMix containing DNase (Tiangen, China). qRT-PCR was conducted using synthetic cDNA, 10 μM of primers, and LightCycler 480 SYBR green I master (Roche, Basel, Switzerland) according to the manufacturer’s instructions. The specific amplification procedure was as follows: 95 °C for 1 min, followed by 40 cycles of three steps (95 °C for 15 s, 55 °C for 30 s, and 72 °C for 15 s), and the 18 S gene was served as housekeeping gene. All samples were independently repeated three times in the plate. The relative mRNA levels of genes were calculated by using comparative ΔΔCt method. The following primer pairs were used. fe-IFN-β-forward (5′-GAAGGAGGAAGCCATATTGGT-3′), fe-IFN-β-reverse (5′-CTCCATGATTTCCTCCAGGAT-3′), fe-IFITM1-forward (5′-CACCACCGTGATCAACATCCA-3′), fe-IFITM1-reverse (5′-GACTTCACGGAGTAGGCAAAG-3′), fe-ISG15-forward (5′-TCCTGGTGAGGAACCACAAGGG-3′), fe-ISG15-reverse (5′-TTCAGCCAGAACAGGTCGTC-3′), fe-Viperin-forward (5′-CATGACCGGGGCGAGTACCTG-3′), fe-Viperin-reverse (5′-GCAAGGATGTCCAAATATTCACC-3′), Fe-18s-forward (5′-CGGCTACCACATCCAAGGAA-3′), Fe-18s-reverse (5′-GCTGGAATTACCGCGGCT-3′).

### 2.5. Coimmunoprecipitation Assays

Briefly, cells were lysed in ice-cold RIPA lysis buffer (Beyotime, Shanghai, China) containing 1 mM phenylmethylsulfonylfluoride (PMSF). The lysates were obtained by centrifugation and incubated with the indicated antibodies at 4 °C overnight on a rotator. Then the cell lysate/antibody immunocomplexes were incubated with Protein G Sepharose beads (Roche) for another 6 h. The beads were washed six times with phosphate buffered saline (PBS) and resuspended in 30–60 μL 1 × SDS loading buffer. The beads were boiled for 10 min at 100 °C to dissociate the immunocomplexes from the beads. SDS-PAGE was performed with the supernatant. Western blot was conducted with the indicated antibodies. The images were collected with the Odyssey infrared imaging system (L1-COR Biosciences, Lincoln, NE, USA).

### 2.6. Statistical Analysis

The data shown represent the means ± SD, and all experiments were repeated three times. Statistical significances were determined using one-way ANOVA with Graphpad software. For all tests * *p* < 0.05, ** *p* < 0.01 and *** *p* < 0.001.

## 3. Results

### 3.1. FIPV-DF2 Infection Interrupts Type I IFN Signaling

**Figure 1:**
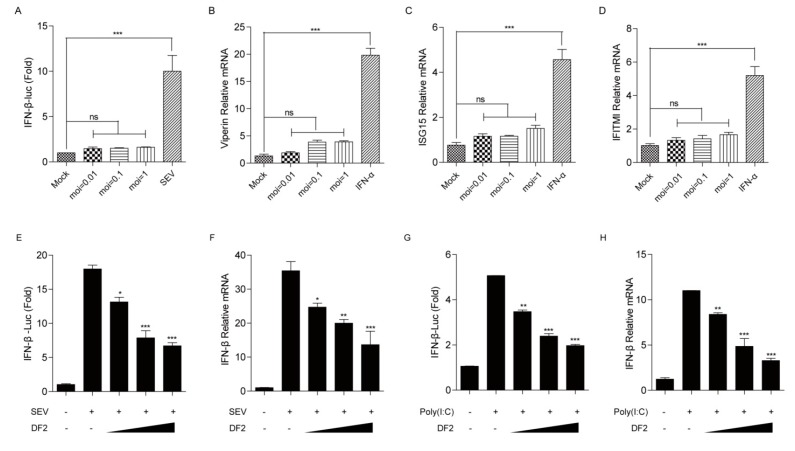
(**A**) CRFK cells were transfected with interferon-β (IFN-β) promoter luciferase reporter plasmid, along with pRL-TK. At 24 hpi, the cells were either mock-infected or infected with increasing concentrations of feline infectious peritonitis virus (FIPV)-DF2 (moi = 0.01, 0.1, 1). The mock-infected cells were infected with 20 hemagglutinating activity units of Sendai virus (SEV) as a positive control. At 12 hpi, the cells were lysed and luciferase assays were performed. (**B**–**D**) CRFK cells were mock-infected or infected with increasing concentrations of FIPV-DF2 (moi = 0.01, 0.1, 1) for 12 h. CRFK cells were treated with IFN-α (500 ng/mL) as a positive control, and the levels of Viperin (**B**), interferon-stimulated gene (ISG)15 (**C**), and IFITMI (**D**) mRNA were detected by qRT-PCR. (**E**,**G**) CRFK cells were co-transfected with IFN-β promoter luciferase reporter plasmid and pRL-TK for 24 h, followed by mock-infection or infection with increasing concentrations of DF2 (moi = 0.01, 0.1, 1) for 12 h and then infection with 20 hemagglutinating activity units of SEV (**E**) or transfection with poly(I:C) (2 μg/mL) (**G**); the cells were lysed and luciferase assays were performed. (**F**,**H**) CRFK cells were mock-infected or infected with increasing concentrations of DF2 (moi = 0.01, 0.1, 1) for 12 h and then infected with 20 hemagglutinating activity units of SEV (**F**) or transfected with poly(I:C) (2 μg/mL) (**H**); the levels of IFN-β mRNA were detected by qRT-PCR. The data shown represent the means ± SD, and all experiments were repeated three times. The significant differences are indicated as follows: * *p* < 0.05, ** *p* < 0.01, *** *p* < 0.001.

The innate immune system provides host protection from infectious diseases. Detection of viral pathogens by innate immune system mainly leads to the induction of innate antiviral mechanisms, most of which are mediated primarily by type-I interferons (IFNs) [40]. To explore whether the FIPV-DF2 infection can activate the host innate immune responses, CRFK cells were co-transfected with the luciferase reporter plasmids IFN-β-luc and pRL-TK, followed by mock-infection or infection with increasing concentrations of FIPV-DF2 as indicated. After 12 h of FIPV-DF2 infection, the IFN-β promoter luciferase reporter system was measured. As shown in Figure 1A, IFN-β promoter activity was barely detectable in DF2-infected cells compared with the strong signal in SEV-infected cells, indicating that FIPV-DF2 infection failed to activate IFN-β promoter activity. Additionally, to further investigate whether FIPV-DF2 infection can activate different ISG expression involved in the IFN signaling pathway, CRFK cells were infected with increasing concentrations of FIPV-DF2 as indicated for 12 h or treated with IFN-α as a positive control, and the levels of the relative ISG mRNA were detected. We found that FIPV-DF2 infection barely induced Viperin (Figure 1B), ISG15 (Figure 1C), and IFITMI (Figure 1D) mRNA levels, although the relative ISGs production were enhanced in IFN-α-pretreated cells. Furthermore, to test whether DF2 infection inhibits SEV-or poly(I:C)-induced IFN-β production, the IFN-β promoter luciferase reporter system and qRT-PCR were used to analyze IFN-β production. As shown in Figure 1E–H, we found that the SEV-or poly(I:C)-induced activation of the IFN-β promoter and the IFN-β mRNA levels were significantly inhibited by FIPV-DF2 infection in a dose-dependent manner. These results suggest that FIPV-DF2 infection impedes type I IFN signaling in CRFK cells.

### 3.2. FIPV nsp5 Inhibits SEV- and Poly(I:C)-Mediated IFN-β Production in a Protease-Dependent Manner

**Figure 2:**
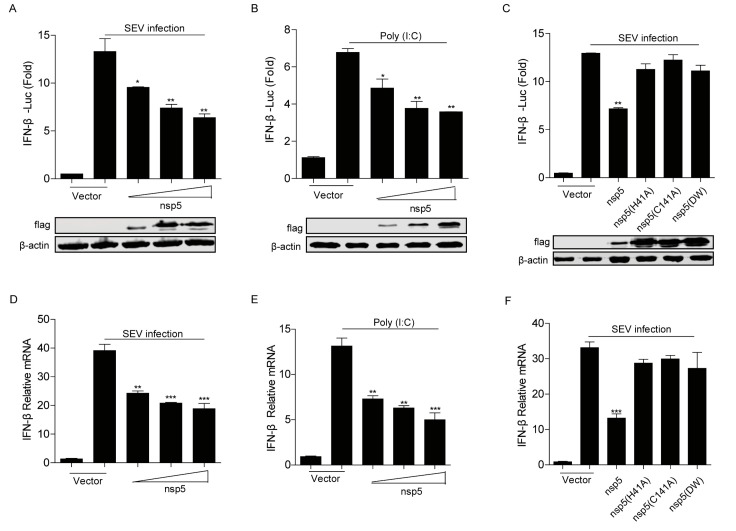
(**A**,**B**) HEK293T cells were co-transfected with IFN-β promoter reporter plasmid and pRL-TK, along with increasing amounts of FIPV-nsp5 (200 ng, 400 ng, 800 ng). At 24 hpi, the cells were infected with 20 hemagglutinating activity units of SEV (**A**) or transfected with poly(I:C) (2 μg/mL) (**B**). At 20 hpi, the cells were collected and the luciferase activities were measured. The expression levels of indicated proteins were analyzed by Western blot. (**C**) HEK293T cells were transfected with IFN-β promoter reporter plasmid and pRL-TK, in combination with a vector expressing the wild-type nsp5, the nsp5 single mutation (H41A, C144A), or nsp5 double mutation (DM) (800 ng), followed by mock infection or SEV infection. At 20 hpi, cell lysates were prepared and the luciferase activities were measured. The expression levels of indicated proteins were analyzed by Western blot. (**D**,**E**) HEK293T cells were transfected with increasing amounts of FIPV nsp5 (200 ng, 400 ng, 800 ng)-expressing plasmid for 24 h and then infected with 20 hemagglutinating activity units of SEV (**D**) or transfected with poly(I:C) (2 μg/mL) (**E**). At 20 hpi, the cells were collected and the mRNA levels of IFN-β were evaluated by qRT-PCR. (**F**) HEK293T cells were transfected with FIPV nsp5 or nsp5 mutants (800 ng) followed by mock infection or SEV infection. At 20 hpi, the cells were collected and the mRNA levels of IFN-β were evaluated by qRT-PCR. The data shown represent the means ± SD, and all experiments were repeated three times. The significant differences are indicated as follows: * *p* < 0.05, ** *p* < 0.01, *** *p* < 0.001.

In this study, to confirm whether FIPV nsp5 inhibits the type I IFN signaling, the effect of FIPV nsp5 overexpression on SEV or poly(I:C) induced IFN-β production in HEK293T cells was evaluated by qRT-PCR and dual luciferase reporter assay. The results showed that FIPV nsp5 inhibited SEV or poly(I:C)-induced IFN-β promoter activation (Figure 2A,B) and its mRNA levels (Figure 2D,E) in a dose-dependent manner. According to a previous report, CoV nsp5 is a cysteine proteinase, which mainly depends on the His41 and Cys144 to conduct its cleavage function. Moreover, nsp5 double-mutant H41A/C144A completely abolishes its protease activity [41]. To further determine whether the antagonistic property of FIPV nsp5 in type I IFN signaling is dependent on its protease activity, three plasmids expressing different mutant versions of FIPV nsp5 (H41A, C144A, H41A/C144A) were constructed and the impact of their expression on SEV induced IFN-β production was evaluated by qRT-PCR and dual luciferase assay. As shown in Figure 2C,F, in contrast to wild-type nsp5, the three nsp5 mutants (H41A, C144A, H41A/C144A) were incapable of suppressing SEV-induced IFN-β promoter activation (Figure 2C) and its mRNA levels (Figure 2F), revealing that the protease activity of FIPV nsp5 is involved in antagonizing IFN-β production.

### 3.3. FIPV Nsp5 Disrupts RLR Signaling by Cleaving NEMO

**Figure 3:**
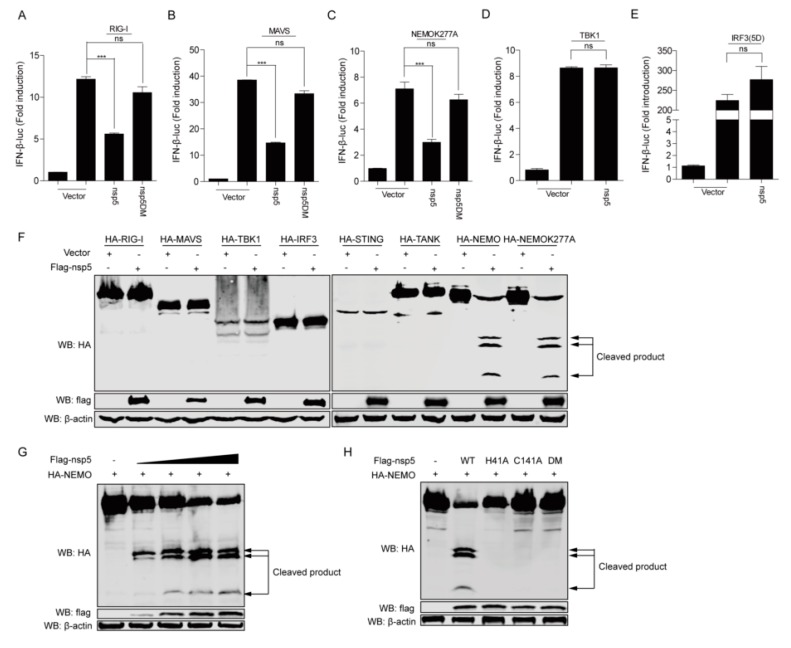
(**A**–**E**) HEK293T cells were transfected with IFN-β promoter luciferase reporter plasmid and pRL-TK, along with a plasmid expressing retinoic acid-inducible gene I (RIG-I) (**A**), mitochondrial antiviral signaling (MAVS) (**B**), NEMOK277A (**C**), TRAF family member-associated nuclear factor κB activator (TANK)-binding kinase 1 (TBK1) (**D**), and interferon regulatory factor 3 (IRF3)(5D) (**E**) (400 ng), as well as FIPV nsp5 or FIPV nsp5DM (400 ng). At 28 hpi, the cells were lysed and luciferase assays were performed. (**F**) HEK293T cells were transfected with a plasmid expressing RIG-I, MAVS, NEMO, NEMOK277A, TANK, stimulator of interferon genes (STING), TBK1, and IRF3 (2 μg), along with FIPV nsp5 or an empty vector (2 μg). At 30 hpi, the cells were harvested for Western blot analysis. (**G**) HEK293T cells were transfected with an expression plasmid NEMO (2 μg), along with increasing amounts of nsp5-expressing plasmids (250 ng, 500 ng, 1 μg, 2 μg). At 30 hpi, the cells were prepared and analyzed by Western blot. (**H**) HEK293T cells were transfected with a plasmid encoding NEMO (2 μg), in combination with plasmid expressing nsp5 or its mutants (nsp5H411A, nsp5C144A, nsp5 double mutation (DM)) (2 μg), The cell lysates were prepared and analyzed by Western blot. NEMO: NF-κB essential modulator. The data shown represent the means ± SD, and all experiments were repeated three times. The significant differences are indicated as follows: ns: no significant difference, *** *p* < 0.001.

To determine adaptors by which FIPV nsp5 exerts its function in RLR-mediated type I IFN production, we examined the effect of FIPV nsp5 overexpression on the IFN-β promoter activation mediated by several crucial feline adaptors in RIG-I/MDA5 signaling, including RIG-I, MAVS, NEMO-K277A (a constitutively active NEMO mutant), TBK1, and IRF3-5D (a constitutively active form of IRF3). The results showed that FIPV nsp5 significantly inhibited IFN-β promoter activation induced by RIG-I, MAVS, and NEMO-K277A (Figure 3A–C), but did not inhibit the activation induced by TBK1 and IRF3-5D (Figure 3D–E), suggesting that FIPV nsp5 inhibited upstream of TBK1. To further investigate which adaptor related to IFN signaling pathways was cleaved by FIPV nsp5, HEK293T cells were transfected with a HA-tagged feline RIG-I, MAVS, NEMO, NEMOK277A, TANK, STING, TBK1, or IRF3 expression plasmid along with an empty vector or a plasmid encoding Flag-tagged FIPV nsp5. As shown in Figure 3F, NEMO and NEMOK277A were cleaved by FIPV nsp5, but no cleavage products were observed in other adaptor proteins. PEDV and FIPV, two members of the coronavirus, both encode nsp5 proteins with similar structures and functions. Previous research revealed that PEDV nsp5 cleaved both human NEMO and porcine NEMO at glutamine 231(Q231) to produce one cleavage product [41]. Interestingly, in this study, three N-terminal fragments were detected under the co-expression of feline NEMO or feline NEMOK277A and FIPV nsp5. This result indicated that FIPV nsp5 protein possesses the ability to cleave multiple sites of feline NEMO. To further confirm whether NEMO was cleaved by FIPV nsp5 in a dose-dependent manner, HEK293T cells were transfected with NEMO expression plasmid, along with increasing amounts of nsp5 expressing plasmids. The result showed that the levels of NEMO gradually declined and three N-terminal fragments began to appear as FIPV nsp5 expression increased (Figure 3G). Notably, the degree of NEMO cleavage was positively correlated with the expression levels of FIPV nsp5. We next determined if NEMO cleavage by FIPV nsp5 was dependent on its protease activity. NEMO was transfected into HEK293T cells, along with a plasmid expressing FIPV nsp5, nsp5H41A, nsp5C144A, nsp5DM (nsp5H41A/C144A) or empty vector. Whereas FIPV nsp5 cleaved NEMO and produced three N-terminal fragments, none of the FIPV nsp5 mutants did so (Figure 3H). This result is consistent with the fact that our earlier observation that the protease activity of FIPV nsp5 is involved in antagonizing the SEV-induced IFN-β production (Figure 2C,F).

### 3.4. NEMO is Cleaved at Gln-132, Gln-205, Gln-231 by FIPV-nsp5

**Figure 4:**
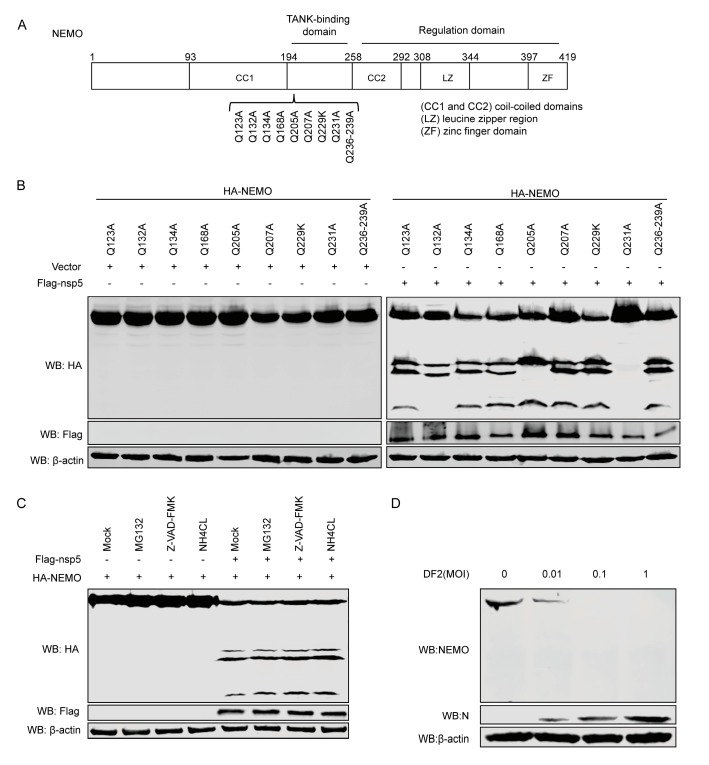
(**A**) Schematic of feline NEMO and its substitution mutants. (**B**) HEK293T cells were transfected with NEMO or NEMO mutants as indicated, along with empty vector or FIPV nsp5. At 30 hpi, cell lysates were prepared and analyzed by Western blot. (**C**) HEK293T cells were transfected with a plasmid expressing NEMO, along with a plasmid expressing FIPV nsp5 or empty vector. At 24 hpi, the cells were pretreated with MG132 (20 μm), Z-VAD-FMK (80 μm), or NH4CL (10 mM) for another 10 h, and then the lysates were analyzed by Western blot. (**D**) CRFK cells were either mock-infected or infected with increasing concentrations of FIPV DF2 (moi = 0.01, 0.1, 1), respectively. After 24 h, the whole cell lysates were analyzed by western blot with anti-NEMO antibody (1:100).

NEMO is an essential component of the IKK complex which contains many distinct domains, including a TANK-binding domain [30], two coil–coiled domains (CC1 and CC2), a leucine zipper region, and a zinc finger domain [32]. Previous studies demonstrated that CoV nsp5 preferentially cleaved glutamine (Gln) at the P1 position [42,43]. In the experiment described above, co-expression of FIPV nsp5 and feline NEMO resulted in the appearance of three N-terminal fragments. Two of them had molecular masses of approximately between 25 and 35kDa, while a smaller new band had a molecular mass of approximately 20 kDa. According to these data, we speculated the amino acid sequence of NEMO for potential FIPV nsp5 cleavage sites which could be able to produce fragments of the appropriate size mentioned above. We inferred that potential FIPV nsp5 cleavage sites existed at amino acids 123–240 within feline NEMO. To examine FIPV nsp5 cleavage sites, we constructed a series of NEMO mutants in which glutamine (Gln) at the P1 position was replaced with alanine or arginine (Figure 4A). HEK293T cells were transfected with NEMO or NEMO mutation, in combination with the expressing plasmid nsp5. As shown in Figure 4B, we observed that at least one cleaved fragment was absent in the NEMOQ132A, NEMOQ205A, or NEMOQ231A/FIPV nsp5 co-expression samples; in contrast, FIPV nsp5-mediated NEMO cleavage was not affected by Q123A, Q134A, Q168A, Q207A, Q229R, or Q236–239A. We further investigated whether cellular caspase activity or proteasome or lysosome signaling are required for FIPV nsp5-mediated NEMO cleavage. HEK293T cells expressing NEMO and nsp5 were treated with a pan-caspase inhibitor (Z-VAD-FMK), proteasome inhibitor (MG132), or lysosome inhibitor (NH4CL), respectively. The results showed that none of the three inhibitors prevented FIPV nsp5-mediated NEMO cleavage (Figure 4C). Moreover, to test whether endogenous NEMO was cleaved during FIPV-DF2 infection, CRFK cells were either mock-infected or infected with increasing concentrations of DF2, and the levels of endogenous NEMO were investigated. As shown in Figure 4D, upon infection with increasing concentrations of DF2, the levels of endogenous NEMO decreased in a dose-dependent manner compared with mock-infected cells.

### 3.5. FIPV nsp5-Mediated NEMO Cleavage Is Involved in the Suppression of IFN-β Induction

**Figure 5:**
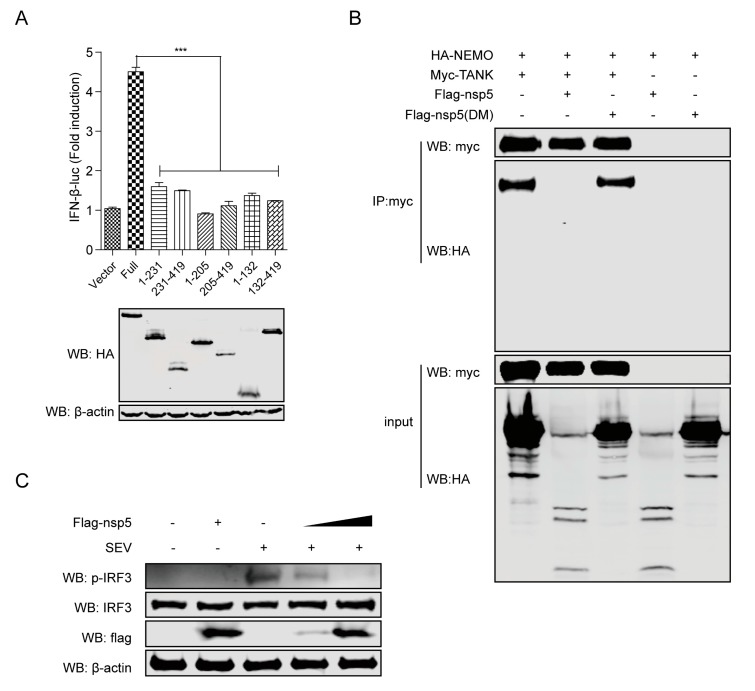
(**A**) HEK293T cells were co-transfected with IFN-β-luc and pRL-TK, in combination with flag-tagged NEMOK277A (full) expression plasmid or NEMOK277A deletion mutants (NEMOK277A-1-205, NEMOK277A-205-419, NEMOK277A-1-231, NEMOK277A-231-419) (800 ng). Luciferase assays were performed at 30 h after the transfection. The expression levels of indicated proteins were analyzed by western blot. (**B**) HEK293T cells were transfected with a plasmid expressing HA-tagged NEMO (1 μg), along with a plasmid expressing Myc-tagged TANK or a vector plasmid (1 μg) and in combination with a plasmid expressing flag-tagged nsp5 or flag-tagged nsp5DM (2 μg). The cell lysates were immunoprecipitated with anti-Myc antibody. The cell lysates and the immunoprecipitants were analyzed by immunoblotting using anti-HA (1:3000) or anti-Myc antibodies (1:3000). (**C**) CRFK cells were transfected with increasing amounts of nsp5-expressing plasmids (0, 1.5 and 3 μg) for 24 h and then infected or not with SEV for another 20 h, and the lysates were then subjected to immunoblotting with anti-phospho-IRF3 (1:100), anti-IRF3 (1:100), anti-Flag (1:3000), and anti-β-actin (1:3000) antibodies. The data shown represent the means ± SD, and all experiments were repeated three times. The significant differences are indicated as follows: *** *p* < 0.001.

NEMO played an important role in IFN signaling pathways. As mentioned above, the activation of the IFN-β promoter induced by NEMOK277A was significantly inhibited in the presence of FIPV nsp5 (Figure 3C). Our earlier observation showed that NEMOK277A was capable of being cleaved by nsp5 (Figure 3F), revealing that the K277A substitution did not alter the susceptibility of NEMO to nsp5. We speculated that FIPV nsp5-mediated cleavage of NEMOK277A likely debilitated the ability to induce IFN-β expression. To examine this hypotheses, we constructed a series of truncated mutants based on the identified nsp5 cleavage sites from NEMOK277A (NEMOK277A-1-132, NEMOK277A-132-419, NEMOK277A-1-205, NEMOK277A-205-419, NEMOK277A-1-231, NEMOK277A-231-419) to assess whether FIPV nsp5-mediated NEMO cleavage fragments retain the ability to induce IFN-β production. As shown in Figure 5A, none of these six truncated mutants were competent in inducing IFN-β promoter activation in comparison with full length NEMOK277A. According to a previous report, NEMO was in charge of physically interacting with TANK and recruiting TBK1 and IKKε to the RIG-I–MAVS complex in the process of virus-induced activation of IRF3 and IRF7 [32]. A NEMO mutant with internal deletion of residues 196–250, which removes the TANK-binding domain [30], failed to associate with TBK1 or IKKε in cells co-expressing TANK [32]. To elucidate whether cleavage of NEMO by FIPV nsp5 attenuated the formation of NEMO–TANK–TBK1 complex, HA-tagged NEMO and Myc-tagged TANK were co-expressed with flag-tagged nsp5 or flag-tagged nsp5DM in HEK293T cells. As shown in Figure 5B, Myc-tagged TANK was immunoprecipitated with HA-NEMO in the absence of FIPV nsp5 and in the presence of nsp5DM (in the panel 1 and 3), whereas the interaction between TANK and NEMO was attenuated in the presence of FIPV nsp5 because of the cleavage of NEMO caused by nsp5 (in panel 2). As phosphorylation is the hallmark of IRF3 activation [44], we next explored the effect of overexpressing nsp5 on the phosphorylation of IRF3. As expected, the total protein levels of IRF3 were almost equal and the SEV-induced IRF3 phosphorylation levels were significantly reduced by nsp5 in a dose-dependent manner (Figure 5C). Taken together, these results indicated that, by disrupting the interaction between TANK and NEMO, the nsp5-mediated NEMO cleavage inhibited the IRF3 phosphorylation and was involved in the suppression of type I IFN production.

## 4. Discussion

FIP is a deadly disease that affects both domestic and wild cats and is caused by virulent feline coronavirus (FCoV). The secretion of type I IFN, IFN-α, and IFN-β is the foundation of the innate host defense against virus infection, followed by the activation of autocrine and paracrine signaling, which induces IFN-stimulated genes (ISGs) with antiviral activities. RIG-I and MDA5 play nonredundant roles in cytosolic RNA sensing by recognizing different groups of viral RNAs. During coevolution with its host, viruses evolve a variety of strategies to evade host innate immune responses. However, it is unclear whether type I IFN responses are inhibited by FIPV. In this study, we found that FIPV-DF2 infection barely induced and significantly inhibited SEV-induced IFN-β production (Figure 1A–H).

To date, the control of FECV replication (which is the source of FIPV-causing mutant virus [45]) and the development of anti-coronavirus drugs [5,23,46] are two major preventive and therapeutic measures of FIPV. Since the coronavirus nsp5-encoded 3C-like protease (3CLpro) plays an important role in virus replication and immune evasion, 3CLpro is considered an attractive target for the development of anti-coronaviral therapeutics. It has been demonstrated that the cleavage of adaptors involved in the signaling pathway by a main viral protease is considered as a particularly effective way for virus to escape the innate immune response. Previous studies reported that the EV71 virus inhibits Toll-like receptor 3-mediated antiviral responses through the cleavage of TRIF and IRF7 by 3Cpro [47,48]. Recently, Li Huang et al. [49] also found that EMCV 3C protease relieved the TANK inhibitory effect on TRAF6-mediated NF-κB signaling through cleavage of TANK. Additionally, NEMO, which is a key adaptor involved in multiple signaling pathways, has been reported to be cleaved by FMDV, HAV, PEDV, and PRRSV [41,50,51,52]. Here, we verified that, similarly to PEDV nsp5, FIPV nsp5 could also disrupt RLR signaling by targeting the crucial feline adaptor NEMO (Figure 3F). Furthermore, we identified that FIPV nsp5, rather than FIPV nsp5DM, is capable of cleaving feline NEMO in a dose-dependent manner (Figure 3G–H), which explains our earlier observation that the protease activity of FIPV nsp5 is involved in antagonizing SEV-induced IFN-β production (Figure 2C,F).

Previous research demonstrated that PEDV nsp5 regulated its interferon antagonism by cleaving porcine NEMO at a single site, Q231, concomitant with the same human NEMO cleavage products [41]. Interestingly, in this study, three N-terminal fragments were detected under the co-expression of feline NEMO or NEMOK277A and FIPV nsp5 (Figure 3F), indicating that FIPV nsp5 protein possesses the ability to cleave multiple sites of NEMO. The size of the largest fragment in FIPV nsp5-medated cleavage was consistent with that in PEDV nsp5-mediated cleavage, while the two new potential FIPV nsp5 cleavage positions were identified as NEMOQ132 and NEMOQ205 (Figure 4B). Surprisingly, we found that NEMOQ231A completely prevented FIPV nsp5-mediated cleavage. The protease–substrate interaction is the first step of the proteolytic activity of proteases. We inferred that the NEMOQ231A substitution makes it lose the ability to bind the protease and form the complex. NEMO contains many distinct domains, including TANK-binding domain [30], two coil–coiled domains (CC1 and CC2), a leucine zipper region, and a zinc finger domain [31,32]. Q132 is located within coil-coiled domain 1, while Q205 and Q231 are located within the TANK-binding domain. Furthermore, we assessed the cleavage of NEMO in FIPV-infected CRFK cells. Unfortunately, we failed to detect the endogenous cleavage products of NEMO in FIPV-infected CRFK cells, but we observed a gradual reduction of the intensity of NEMO following FIPV infection (Figure 4D). Similar situations were also reported in previous studies. SVV 3Cpro could cleave multiple sites of MAVS, TRIF, and TANK and produced the corresponding cleavage products, despite cleavage products not being observed in SVV infected cells [53]. We inferred that the cleavage products may be short-lived during FIPV-nsp5 infection.

NEMO, which serves as a regulatory subunit of IKK complex, plays an essential role in RNA virus-induced activation of IRF3 and IRF7. It has been reported that expression of MAVS or the constitutively active RIG-IN in NEMO−/− MEFs failed to stimulate phosphorylation and activation of IRF3 and IRF7 [31,32]. A recent study demonstrated that deletion of 150 amino acids from the N-terminal which removed the partial coiled–coiled domain 1 completely abrogated SV and MAVS-induced ISRE-luciferase activity [31,32]. Additionally, The association of NEMO with TANK facilitated the recruitment of TBK1 and IKKε to the MAVS mitochondrial complex in the process of IRF3 and IRF7 activation, and a NEMO mutant with internal deletion of residues 196–250 [30], which removes the TANK-binding domain, failed to associate with TBK1 or IKKε in cells co-expressing TANK [31,32]. In this study, we also verified that the cleavage fragments generated from targeting these three NEMO sites (Q132, Q205 and Q231) have lost the ability to induce IFN-β production (Figure 5A), indicating that the CC1 domain and TANK-binding domain are indispensable for NEMO-induced IFN signaling. Further investigation revealed that the interaction between TANK and NEMO was attenuated in the presence of FIPV nsp5 (Figure 5B). Moreover, we determined that FIPV-nsp5 inhibits SEV-induced IRF3 phosphorylated (Figure 5C).

**Figure 6:**
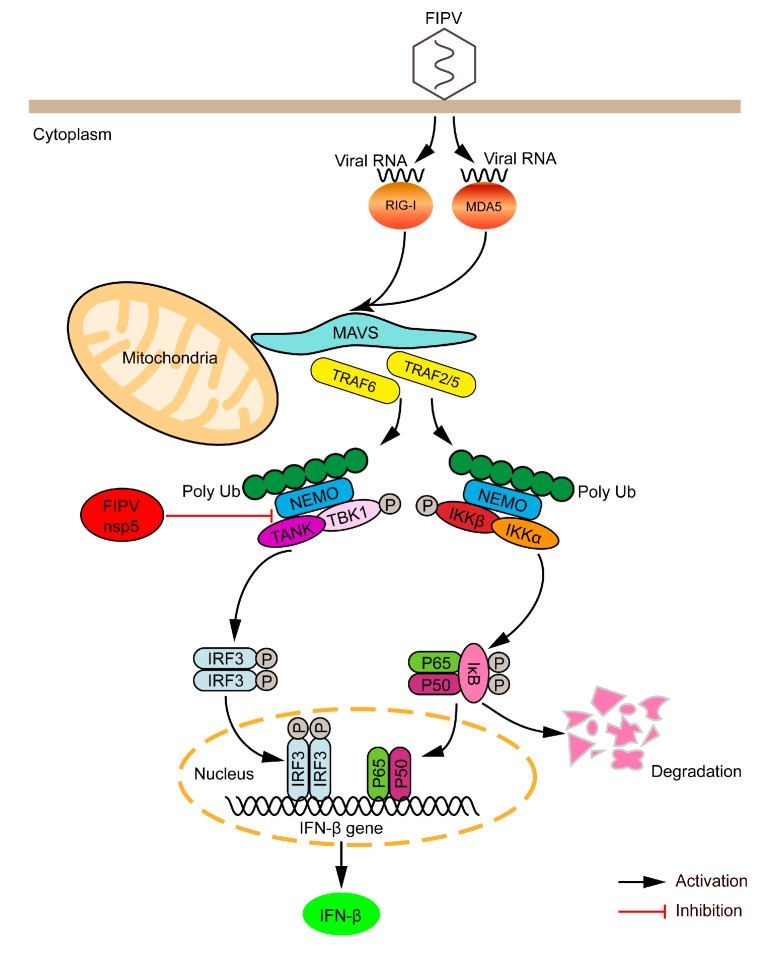
Schematic model of FIPV nsp5-mediated inhibition of the type I IFN signaling pathway during FIPV infection. RIG-I and MDA5 play nonredundant roles in cytosolic RNA sensing by recognizing different groups of viral RNAs. RIG-I and MDA5 activation induce the polymerization of MAVS, which in turn recruits and activates E3 ligases TRAF2, TRAF3, TRAF5, and TRAF6. These E3 ligases then synthesize polyubiquitin chains that are sensed by NEMO through its ubiquitin-binding domains. NEMO then interacts with TANK and recruits TBK1 and IκB kinase complex (IKKε) to the MAVS polymer, where the kinases phosphorylate IRF3, leading to the induction of type I IFN. However, the FIPV nsp5-mediated NEMO cleavage disrupted the interaction between TANK and NEMO, which reduced the phosphorylation of IRF3, leading to the inhibition of type I IFN production.

In summary, we report that FIPV nsp5 is a negative regulator of RLR-mediated type I IFN production. Mechanistically, the FIPV nsp5-mediated NEMO cleavage disrupted the interaction between TANK and NEMO, which resulted in inhibition of IRF3 phosphorylation and suppression of type I IFN production (Figure 6). These findings provide a further explanation of how FIPV nsp5 efficiently inhibits host IFN response, and contribute to our understanding of the mechanism of FIPV to evade innate immunity strategies.
